# Dissolution and physical characterization of oral nicotine pouch products

**DOI:** 10.1038/s41598-025-34556-5

**Published:** 2026-01-06

**Authors:** Sean P. Platt, Christa M. Gonzales, Pashupati Pokharel, Seok Chan Park, Akchara Sriram, Steven B. Thorpe, Abaigeal B. Ritzenthaler, Fadi Aldeek

**Affiliations:** Altria Client Services LLC, 601 East Jackson Street, Richmond, VA 23219 USA

**Keywords:** Nicotine pouch, Filler, Dissolution, PH, Particle size, Density, Flowability, Solubility, Oven volatiles, Imaging, Microscopy, Crystallinity, Viscosity, Characterization, Chemistry, Materials science

## Abstract

In this study, we conducted a comprehensive analysis of seven commercially available nicotine pouch products, including *on!*, *Zyn*, *Velo*, *Dryft*, *Rogue*, *Volt*, and *Loop* nicotine pouches. Analyses include nicotine content, nicotine dissolution release, particle size, imaging (bulk filler and outer pouch material), bulk density (tapped and untapped), true density, crystallinity, solubility, oven volatiles (OV as measured by percent moisture content, % MC), pH of aqueous extracts, water activity, and extract viscosity. The nicotine dissolution profiles showed a faster release for *on!*, *Zyn*, and *Rogue* when compared to *Velo*, *Dryft*, *Volt*, and *Loop* nicotine pouches. When all nicotine pouches release profiles were compared to *on!*, only *Zyn* and *Rogue* were found equivalent. Particle size analysis revealed Gaussian-like distributions for *on!*, *Rogue*, and *Zyn* nicotine pouches. The remaining products displayed bimodal particle size distributions. Tapped and untapped bulk densities were measured to assess the flowability of the filler in all seven nicotine pouches. *Rogue* and *Loop* nicotine pouches exhibited the highest and lowest flowabilities, respectively. Solubility data indicated that *Zyn* nicotine pouches had the highest percentage of soluble components, whereas *Rogue* nicotine pouches had the lowest. Rheology results from the nicotine pouch extracts in artificial saliva showed the highest viscosity for *Loop* and lowest viscosity for *Volt* and *Rogue* nicotine pouches. All products were found to have crystalline structures with both high and low temperature melting points. Our results indicate that nicotine pouch products exhibit differences and similarities in their physicochemical properties, providing valuable insights into understanding their formulation and development.

## Introduction

Modern oral nicotine products such as nicotine pouches, lozenges, orbs, sticks, strips, and gums contain nicotine that is either tobacco derived or synthetic. These products do not contain cut, ground, powdered, or leaf tobacco^1–7^. Nicotine pouches or “tobacco-free nicotine pouches” are a growing category of oral products in many parts of the world. In addition to nicotine, nicotine pouches contain a base material, often cellulose based, flavors, sweeteners and sugar alcohols, and pH adjusters. Several brands, such as *on!*, *Zyn*, *Velo*, *Dryft*, *Rogue*, *Volt*, and *Loop*, have been introduced to the market, offering nicotine pouch products with a variety of flavors and nicotine strengths to meet diverse consumer preferences^3^. Chemical characterization methods for the determination of nicotine, nicotine degradants, harmful and potentially harmful constituents (HPHC), and determination of the rate of nicotine release through dissolution testing of nicotine pouches have been studied^1,8–12^. However, there is limited research on the physical properties of nicotine pouches, which may impact the dissolution rate and overall performance of the product^13^.

In this study, seven nicotine pouch products, *on!*, *Zyn*, *Velo*, *Dryft*, *Rogue*, *Volt*, and *Loop*, were extensively characterized using a wide range of chemical and physical methods. These brands were selected to represent both dry (*on!*, *Zyn*, *Velo*, *Dryft*, *Rogue*) and wet (*Volt*, *Loop*) pouch types, including top-selling products in the United States and other representative market offerings. Chemical characterization included nicotine quantitation using gas chromatography with flame ionization detection (GC-FID), following a CORESTA recommended method (CRM), and dissolution testing was conducted using the USP-4 flow-through cell apparatus, coupled with ultra-performance liquid chromatography and photodiode array detection (UPLC-PDA)^11,14–16^. Physical characterization testing included particle size by dynamic image analysis, imaging by optical and scanning electron microscopy (SEM), bulk density (untapped and tapped), true density, crystallinity using differential scanning calorimetry (DSC), solubility, percent moisture content using rapid halogen oven volatiles (OV), pH of aqueous extracts, water activity, and viscosity of extracted solutions. This study establishes a foundational scientific understanding of nicotine pouch products through a comprehensive suite of chemical and physical characterization methods, which is critical for identifying formulation attributes and performance factors that influence nicotine release. These insights enable more informed product development and support innovation within this rapidly growing category.

## Materials and methods

### Nicotine pouch products

Seven (7) nicotine pouch products were used in this study with the product names and manufacturers listed in Table 1. To be consistent, all products were mint flavored with nicotine levels between 6 and 9.4 mg per pouch based on the can label. All products were purchased online at the same time, with two lots per brand used for verification. The manufacturing dates for *on!*, *Zyn*, *Velo*, *Dryft*, and *Rogue* nicotine pouch products, as shown on the product labels, indicated they were approximately four to six months old when received, while *Volt* and *Loop* were about one month old. All testing was conducted within two months of receiving the nicotine pouch products. The cans were kept in ambient conditions and used either as received (intact product consisting of filler in an outer pouch material) or as loose filler where the contents of pouches from multiple cans of a product were emptied and used to create a composite sample.

**Table 1:** Summary of nicotine pouch products used in the study including brand name, flavor, labelled nicotine strength per pouch, and manufacturer.

Brand name	Flavor	Nicotine content per pouch	Manufacturer
*on!*	Mint	8 mg	Helix Innovations LLC
*Zyn*	Cool Mint	6 mg	Swedish Match
*Velo*	Spearmint	7 mg	Reynolds America, Inc.
*Dryft*	Spearmint	7 mg	British American Tobacco
*Rogue*	Spearmint	6 mg	Rogue Holdings LLC
*Volt*	Spearmint Breeze	Strength: 3 (8.3 mg)	Swedish Match
*Loop*	Mint Mania	Strength: 3 (9.4 mg)	Another Snus Factory Stockholm AB

### Chemicals and reagents

Methanol (Optima grade), Type-1 water, acetonitrile, and sodium chloride (anhydrous crystal, ACS grade) were purchased from Thermo Fisher Scientific (Wyman, MA). Potassium phosphate (K_2_HPO_4_·H_2_O, ACS grade, anhydrous), calcium chloride dihydrate (CaCl_2_·2H_2_O), potassium chloride (anhydrous), potassium carbonate (anhydrous), magnesium chloride hexahydrate (MgCl_2_·6H_2_O), hydrochloric acid, 5 N sodium hydroxide solution, and caffeine (> 98.5%) internal standard were purchased from Acros Organics (Geel, Belgium). 6 N ammonium hydroxide was acquired from Ricca Chemicals (Pocomoke, MD). 1 M acetic acid was procured from Fluka Analytical (Pittsburgh, PA). ISO 17,034 certified nicotine solution (10 mg/mL) was purchased from Restek Corporation (Bellfonte, PA). Quinoline internal standard in methanol was acquired from SPEX CertiPrep (Metuchen, NJ). Sodium hydroxide pellets, 2 N sodium hydroxide solution, and methyl *t*-butyl ether (MTBE) were purchased from Thermo fisher Scientific (Wyman, MA). Free base neat nicotine (-)-nicotine was acquired from Sigma-Aldrich (Burlington, MA).

### Nicotine determination

The nicotine content in each nicotine pouch product was determined using the CORESTA recommended method (CRM) No. 62 in triplicate^11^. Briefly, one gram (1 g) of sample was extracted in a mixture of water, sodium hydroxide (NaOH), and methyl *t*-butyl ether (MTBE) solvent with quinoline as the internal standard. After extraction, an aliquot of the organic phase was analyzed by gas-chromatography with flame ionization detection (GC-FID) (Agilent Technologies 7890, Agilent, Wilmington, DE). The GC-FID system was equipped with Agilent Technologies 7693 automatic liquid sampler system with auto-injector, 150-sample tray, and HP-5, cross-linked 5% phenyl methylpolysiloxane 30 m x 0.32 mm x 0.25 μm column (Agilent, Wilmington, DE).

### Nicotine dissolution

Nicotine dissolution testing refers to the evaluation of how nicotine is released from nicotine pouch products into artificial saliva over a specified period of time. The amount of nicotine released from the nicotine pouches over sixty minutes (60 min) in artificial saliva and its rate of release, used to generate a nicotine dissolution release profile for each nicotine pouch product, was studied following FDA guidance for industry using a USP-4 flow-through cell apparatus (SOTAX, Westborough, MA) and twelve (12) replicates for each nicotine pouch product^14^. Nicotine collected in the dissolution fractions was quantified using Acquity I-Class Ultra Performance Liquid Chromatograph coupled to a PDA detector (UPLC-PDA) (Waters, Milford, MA). The UPLC system was equipped with a BEH C18 analytical column (2.1 mm x 100 mm x 1.7 μm) and a BEH C18 VanGuard pre-column (2.1 mm x 5 mm x 1.7 μm) (Waters, Milford, MA). The artificial saliva was prepared following the recipe provided by the German Institute for Standardization (DIN) in DIN V Test Method 53160-1 2002 − 10.^15^ The dissolution testing method was carried our according to previously published work^2,16–18^.

### Nicotine dissolution using a kinetic model

To further confirm the experimental nicotine dissolution data from the nicotine pouch products, a first-order kinetic model was applied to predict the total nicotine content available for release and to estimate the percentage of nicotine released within 60 min period. This first-order kinetics model was also applied to determine the nicotine dissolution release rate from the nicotine pouches. The model was fitted to the cumulative nicotine release profiles obtained from each of the twelve replicates for all seven nicotine pouch products, using:

1 $$\:{M}_{rel}={M}_{0}\left(1-{e}^{-kt}\right)$$

where *k* represents the first-order release rate constant, *M*_*rel*_ denotes the cumulative amount of nicotine measured at each specified time point (4, 8, 12, 16, 20, 30, 40, 50, and 60 min), and *M*_0_ is the nicotine content at time zero (*t* = 0). Each replicate’s cumulative nicotine release profile was fitted to Eq. 1.

### Particle size analysis – dynamic image analysis

Particle size analyses were carried out using dynamic image analysis (DIA) conforming to ISO standards for DIA and graphical representations^19,20^. A half teaspoon (0.5 tsp) sample of nicotine pouch filler from a composite sample was introduced on a vibrating sample tray (VIBRI) through a gravity fall dry dispersion module (GRADIS) fitted with a four (4.0) mm cuvette into the DIA system (QICPIC) (Sympatec, Pennington, NJ). The system was equipped with a pulsed laser light source and camera system with a M8 lens with a measuring range of 10–6820 μm and sensor that captured images at a 25 Hz frame rate. The captured images were analyzed to produce particle size distributions and statistical values of the distributions using WINDOX software (version 5.10.0.1) (Sympatec, Pennington, NJ). Result outputs were calculated as the equal projection of a circle (EQPC) with distributions (*q*_n_) weighted by volume and count (*n* = 3 and 0). For each distribution, the mean and intercepts at 10%, 50% (also the median for distributions with a single mode), and 90% of the cumulative distributions (*d*_10_, *d*_50_, and *d*_90_) were calculated. The width of the distribution, or the span, was calculated using the cumulative distribution percent values with Eq. 2.

2 $$\:\mathrm{S}\mathrm{p}\mathrm{a}\mathrm{n}=\frac{\left({d}_{90}-{d}_{10}\right)}{{d}_{50}}$$

The distribution span serves as an indicator of particle size consistency where the closer the value is to 0, the more uniform the distribution and consistent the particle size.

### Microscopy, imaging, and elemental analysis

The morphology and size distribution of the filler and outer pouch material of the nicotine pouch products were assessed using optical microscopy and scanning electron microscopy (SEM).

Optical microscopy was performed using a VK X-3000 confocal laser scanning microscope (CLSM) (Keyence Elmwood Park, NJ). Images were acquired with combined coaxial and ring lighting using Keyence’s focus variation mode which performs a z-stack acquisition to ensure all aspects of the micrograph are in focus.

SEM was conducted using a JEOL LSM IT-700-HR field emission SEM with an energy dispersive X-ray (EDS) detector (JEOL, Peabody, MA). SEM micrographs were acquired with an accelerating voltage of one (1) kV, a probe current of 50, and approximate working distance of 10.5 mm^21^. Furthermore, elemental analysis was obtained using energy dispersive X-ray spectroscopy (EDS) to better understand the different compositions of the filler material used in the seven nicotine pouch products^21,22^. EDS spectra were collected with an accelerating voltage of 20 kV, a probe current of 50, and approximate working distance of 10.5 mm.

For sample preparation, the pouch filler was applied to a 12.5 mm x 10 mm aluminum SEM stub using double sided carbon tape with excess filler removed using canned air. Because *Volt* pouch filler contains a high amount of OV, obtaining a clear image of the filler as received was not possible. Therefore, before imaging, the *Volt* pouch filler was dried in a vacuum oven for three days to facilitate micrograph acquisition of the particles. In contrast, fillers from all other products were imaged directly as removed from their respective pouches. All other product fillers were taken directly from pouches. For micrographs of the pouch material, the material of emptied pouches was rinsed with DI water and dried overnight at ambient conditions.

### Density, flowability, and porosity

The density of the nicotine pouch filler was measured by three (3) separate methods, untapped and tapped bulk density and true density by helium pycnometry. Bulk density represents the loose (untapped) and mechanically compacted (tapped) forms of the filler. The true density is the density of the filler material itself, excluding any pores or void spaces. The untapped and tapped bulk density values were measured using the United States Pharmacopeia (USP) < 616 >^23^.

For untapped bulk density (*ρ*_UB_), the nicotine pouch filler was slowly poured through a 10 mm screen to remove agglomerates, and the filler was allowed to freely flow through a Scott volumeter (Gardner Company, Inc/, Columbia, MD). Once the 25 mL cup, included with the volumeter, began to overflow with filler, the excess was removed from the top and sides. The mass of the filler in the cup was weighed to quantify the untapped bulk density in 25 mL.

For tapped bulk density (*ρ*_TB_), 100 g of filler was weighed and placed in a 250 mL glass graduated cylinder. The cylinder was secured onto a tapped density test stand (SVM II Tapped Density Tester) (Erweka, Newton, PA) and the initial volume was recorded. Samples were tapped for an initial 500 taps then another 750 taps with the volume recorded at each step. Testing was complete if the difference between the volumes of two sets of taps was less than 2% (2%); if the difference was greater, an additional 500 taps were performed. The tapped density was calculated by dividing the mass of the filler by the volume recorded after the final set of taps.

The true density (*ρ*_T_) of all nicotine pouch filler samples was determined by gas pycnometry using a Ultrapyc 5000 true density analyzer (Anton-Paar USA, Inc. Ashland, VA) with helium as the gas used^24^. The nicotine pouch filler was transferred into the 10 cm^3^ sample cell and filled to approximately 75% of the sample cell with the mass recorded. The sample cell and reference chamber were purged by flowing helium gas for 2 min prior to each measurement. After the pressure in the reference chamber stabilized at a predefined pressure, 10 PSI, a valve that connects the reference chamber to the sample cell, was opened. The pressure of the expanded helium gas was measured once it stabilized. The system temperature was controlled at 25 °C throughout the measurement. The gas pycnometer measured the pressure change of the pressurized helium gas filling a volume-calibrated reference chamber when it expanded into a second volume-calibrated chamber containing a sample of known mass. The resulting pressure readings were used to calculate the volume of the sample (*V*_sample_) using the equation:

3 $$\:{V}_{\mathrm{s}\mathrm{a}\mathrm{m}\mathrm{p}\mathrm{l}\mathrm{e}}\:=\:{V}_{\mathrm{c}\mathrm{h}\mathrm{a}\mathrm{m}\mathrm{b}\mathrm{e}\mathrm{r}}\:+\:{V}_{\mathrm{r}\mathrm{e}\mathrm{f}\mathrm{e}\mathrm{r}\mathrm{e}\mathrm{n}\mathrm{c}\mathrm{e}}\left(1\:-\:\frac{{P}_{1}}{{P}_{2}}\right)$$

where *P*_1_ is the pressure of the reference chamber pressurized with helium gas to a predefined value, *P*_2_ is the pressure of the system when the pressurized gas expands into the sample chamber, *V*_chamber_ is the volume of the sample chamber, and *V*_reference_ is the volume of the reference chamber^25,26^. Each measurement conducted up to five (5) runs and stopped when the percent variance of three (3) successive runs was lower than 0.05%. The results from those three (3) runs were averaged to determine the true density of the sample. If the percentage variance of any three successive runs did not meet this criteria, the final three values of the five runs were used to meet the 0.05% criteria.

The untapped bulk density (*ρ*_UB_), tapped bulk density (*ρ*_TB_), and true density (*ρ*_T_) results were further used to calculate the flowability and porosity percentage of the nicotine pouch fillers. The flowability of the nicotine pouch was determined using the Carr’s compressibility index (CI) and Hausner ratio (HR). The CI is calculated using the equation:

4 $$\:CI=\:\frac{\left({\rho\:}_{\mathrm{T}\mathrm{B}}-{\rho\:}_{\mathrm{U}\mathrm{B}}\right)}{{\rho\:}_{\mathrm{TB}}}\times\:100$$

where the closer the untapped and tapped bulk density values are the smaller the CI and the freer flowing the nicotine pouch filler. The HR was calculated using the equation:

5 $$\:HR=\:\frac{{\rho\:}_{\mathrm{T}\mathrm{B}}}{{\rho\:}_{\mathrm{U}\mathrm{B}}}$$

where HR greater than 1.25 is considered poor flowing nicotine pouch filler. The porosity percent (*P*%) combines the tapped bulk and true density to estimate the total void space of open and closed pores using the Eq. 6 where all other variables are previously defined.

6 $$\:P\%=\frac{\left({\rho\:}_{T}-{\rho\:}_{\mathrm{T}\mathrm{B}}\right)}{{\rho\:}_{T}}\times\:100$$

### Crystallinity determination

The degree of structural order in filler materials (also known as crystallinity), could play a role in nicotine pouch performance. Differential Scanning Calorimetry (DSC) provides melting point and enthalpy data, indicating thermal stability and guiding manufacturing processes such as drying and storage. For this, the crystallinity of the seven nicotine pouch filler was determined by DSC following ASTM D3418-21^27^. Briefly, six to ten milligrams (6–10 mg) of filler material was removed from a pouch and placed in a Tzero pan and then precisely sealed. A Discovery DSC 2500 (TA Instruments, New Castle, DE) and an empty Tzero pan as the reference was used for all thermograms using a temperature range from 0 °C to 160 °C with a heating ramp of 10 °C/min. Analysis was performed using TRIOS v. 5.6.0.87 (TA Instruments, New Castle, DE).

### Solubility in artificial saliva

The percent soluble and insoluble materials of nicotine pouch products in artificial saliva (simplified as solubility for the purposes of this study) was determined by shaking one gram (1 g) of pouch filler (*m*_Filler_) in 40 mL artificial saliva for 10 min at 150 rpm using a Innova 2100 platform shaker (New Brunswick Scientific, Edison, NJ). The mixture was then centrifuged for 10 min at 1500 rpm using a Sorvall ST 40R centrifuge (Thermo Scientific, Waltham, MA). Samples were filtered through pre-weighed Whatman #1 filter papers (*m*_Paper_) to collect insoluble material (Whatman, Maidstone, UK). The filter paper and collected material were dried at 120 °C for one (1) hour in an Isotemp oven (Fisher, Pittsburgh, PA) before being transferred to a desiccator to cool for at least 20 min. The dried material (the material that was insoluble in artificial saliva) was weighed including the filter paper and the mass (*m*_Final_) was recorded. The final percentage of soluble materials was calculated using Eqs. 7,

7 $$\:\mathrm{\%}\:\mathrm{S}\mathrm{o}\mathrm{l}\mathrm{u}\mathrm{b}\mathrm{l}\mathrm{e}\mathrm{s}=\:\frac{{m}_{\mathrm{F}\mathrm{i}\mathrm{n}\mathrm{a}\mathrm{l}}-{m}_{\mathrm{P}\mathrm{a}\mathrm{p}\mathrm{e}\mathrm{r}}}{{m}_{\mathrm{F}\mathrm{i}\mathrm{l}\mathrm{l}\mathrm{e}\mathrm{r}}}\times \:100$$

### Rapid OV

To distinguish between dry and wet nicotine pouches in this study, oven volatiles (OV) expressed as a percent of moisture content (% MC) was measured using a HE53 halogen moisture analyzer (Mettler Toledo, Columbus, OH) where two grams (2 g) of filler from a composite sample was weighed on aluminum sample pans (Mettler Toledo, Columbus, OH) using the balance within the analyzer. The sample was run at 80 °C for 10 min in triplicate to record the %MC.

### pH of aqueous extracts

The pH of the aqueous extracts for all nicotine pouch products was determined using a SevenMulti pH meter fitted with a pH Sensor InLab Expert Pro-ISM probe (Mettler Toledo, Columbus, OH) following the Center for Disease Control (CDC) method for pH in smokeless tobacco products^28^. For *on!*, four (4) pouches were used while for all other products two (2) pouches were used for all measurements to hit a target weight between 1.0 and 1.5 g. Each pouch was cut in half with the filler content emptied into 20 mL of Type-1 water along with the cut pouch. The mixture was stirred with a magnetic stirring bar continuously throughout the measurement with the pH of the aqueous extract and temperature values recorded once the submerged probe stabilized. Three replicates were run for each nicotine pouch product.

### Water activity

Water activity (a_w_) is defined as the measure of the amount of unbound water available in a material (not the total amount of water). It is also known as the ratio of the vapor pressure of water in the product to the vapor pressure of pure water at the same temperature. Water activity in the nicotine pouch products was measured using an Aqualab TDL (Tuneable Diode Laser) Water Activity Meter (Pullman, WA). Before analyzing samples, verification standards with water activity (aw) values of 0.250, 0.500, 0.760, and 0.920 were tested to confirm instrument accuracy. Each pouch was placed in a plastic sample cup and inserted into the TDL test chamber. The test was conducted at 25 °C until water activity stabilized, and the value was recorded. All samples were analyzed in triplicate for each product.

### Viscosity of extracted solutions

Nicotine pouches vary by manufacturer and formulation, with differences in ingredients that can influence extract solubility in artificial saliva and solution viscosity. These factors affect nicotine dissolution, diffusion from the pouch, and delivery to the oral cavity^29^. Therefore, evaluating the viscosity of extracted solutions is essential for understanding the dissolution profiles of nicotine pouch products. The viscosity analysis was conducted by mixing four grams (4 g) of nicotine pouch filler from a composite sample with 36 g of artificial saliva in a 50 mL centrifuge tube. The tube was then placed in a Geno/Grinder for 30 min at 1500 rpm. The mixture was then placed in a centrifuge from Thermo Fisher Scientific Sorvall ST40R for 30 min at 4000 rpm. The supernatant (extracted filler) was filtered using a Whatman filter paper with a 0.45 μm pore size. The viscosity of 25 mL of the supernatant was then measured using a Discovery HR30 Rheometer (TA Instruments, New Castle, DE) using a DIN concentric cylinder Peltier aluminum accessories from a shear rate of 1–100 1/s at a geometry gap of 5917.1 μm. Data analysis was carried out using TRIOS v. 5.6.0.87 (TA Instruments, New Castle, DE).

## Results

### Nicotine determination and dissolution testing

The total nicotine level in each of the seven pouch products listed in Table 1 were determined using the CORESTA Recommended method (CRM) No. 62 in triplicate^11^. This method has been standardized for a variety of tobacco products and has recently been proven suitable for analyzing nicotine pouch products. The results for all seven nicotine pouch products in this study are shown in Table 2 with the labeled and calculated milligram (mg) per pouch concentrations. Six of these pouch products, *on!*, *Zyn*, *Velo*, *Dryft*, *Rogue*, and *Volt* were labeled with a target mg/pouch concentration on the can. The nicotine level of *Loop* was taken from NicoLeaks^30^. For *on!*, *Rogue*, and *Volt* nicotine pouches, the calculated nicotine content was slightly higher than the target, measuring 8.2 mg/pouch for *on!*, 6.2 mg/pouch for *Rogue*, and 8.9 mg/pouch for *Volt* versus the 8 mg/pouch, 6 mg/pouch, and 8.3 mg/pouch labeled on the can. The nicotine content in *Zyn* and *Loop* pouches was found to be 5.4 mg/pouch and 9.1 mg/pouch, slightly below the target of 6 mg/pouch and 9.4 mg/pouch. However, the calculated nicotine content for *Velo* and *Dryft* (both labeled as 7 mg) was significantly lower than the target, measuring 4.6 mg/pouch and 5.7 mg/pouch, respectively.

**Table 2:** Quantified and estimated nicotine results of nicotine pouch products. Quantified results include pouch weight (*n* = 12), labeled nicotine content, experimental nicotine content in mg/pouch based on GC-FID results (*n* = 3), and percentage nicotine released in 60 min from dissolution (*n* = 12). Results from a first-order kinetic model based on estimated nicotine release include the initial nicotine content (*M*_*0*_), estimated dissolution release rate constant (*k*) in min^−1^, and estimated percentage nicotine released in 60 min.

Product	Quantified results				Estimated results		
	Pouch Weight (g) (*n* = 12)	Labelled Nicotine (mg/pouch)	Experimental Nicotine Content (mg/pouch (*n* = 3)	Percent (%) Nicotine Released from Content (*n* = 12)	Estimated Nicotine Content at M_0_ (mg/pouch)	Calculated Dissolution Release Rate Constant, k (min^− 1^)	Estimated Percent (%) Nicotine Released from Content
*on!*	0.25 ± 0.009	8	8.2 ± 0.07	98 ± 4	8.380	0.080	97
*Zyn*	0.38 ± 0.002	6	5.4 ± 0.03	96 ± 2	5.350	0.103	97
*Velo*	0.33 ± 0.043	7	4.6 ± 0.54	88 ± 7	4.297	0.057	94
*Dryft*	0.37 ± 0.015	7	5.7 ± 0.88	90 ± 7	5.695	0.042	90
*Rogue*	0.69 ± 0.043	6	6.2 ± 0.01	72 ± 5	4.595	0.078	97
*Volt*	0.64 ± 0.022	8.3	8.9 ± 0.01	89 ± 3	8.225	0.056	96
*Loop*	0.60 ± 0.024	9.4	9.1 ± 0.01	38 ± 2	4.740	0.021	72

**Fig. 1:**
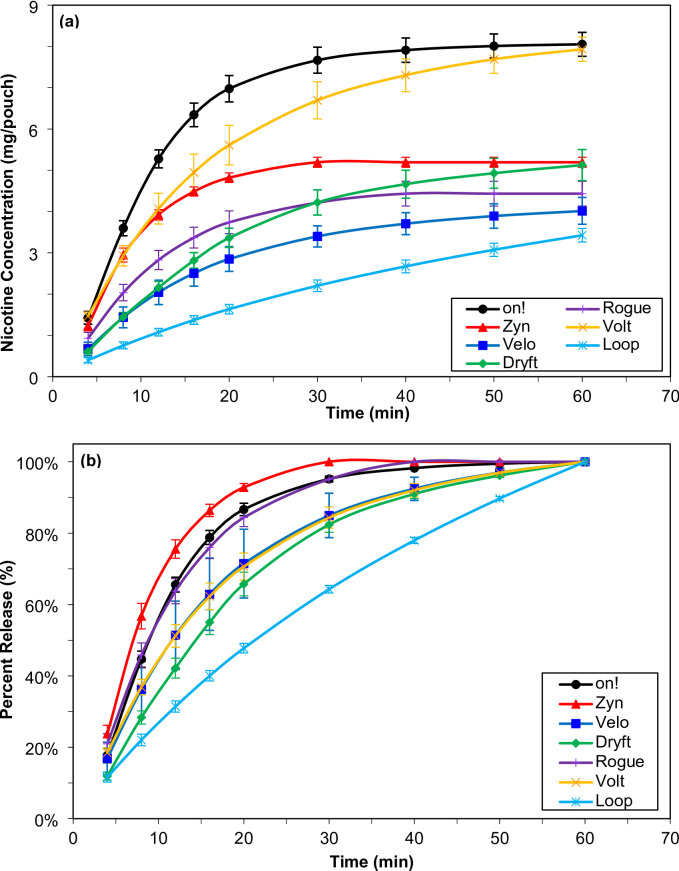
Experimental (**a**) cumulative dissolution release and (**b**) percent of total release profiles of nicotine collected from seven nicotine pouch products (*n* = 12). Error bars denote one standard deviation (1 σ).

To understand the nicotine dissolution release rate from these nicotine pouches, dissolution testing was performed following the FDA guidance for industry using a well-established methodology validated for smokeless tobacco and nicotine pouch products^2,16,18^. Figure 1(a) shows the cumulative nicotine dissolution release profiles on a per pouch basis. The amount of nicotine released from the nicotine pouch is dependent on the nicotine content within each pouch. Table 2 shows the percentage of total nicotine released in 60 min of experiment time based on the amount of nicotine determined in each pouch. Data show that while over 88% of nicotine was released from *on!*, *Zyn*, *Velo*, *Dryft*, and *Volt* nicotine pouches under the same experimental conditions within 60 min, only 72% and 38% of nicotine was released from *Rogue* and *Loop* pouches, respectively. This observation suggests that differences among these pouches affect their ability to dissolve and deliver nicotine, as higher nicotine content does not result in a higher percentage of release.

The cumulative nicotine dissolution release profiles were normalized to better understand the nicotine release rate from these pouches. For this, the nicotine, as a percentage of total release, was calculated and values were plotted versus time as shown in Fig. 1(b). The percentage of total release was calculated by dividing the amount of nicotine released at each time for each fraction by the cumulative amount released at 60 min. By normalizing the data, product-to-product comparison of nicotine pouches can occur. The highest release profile was obtained for *Zyn* nicotine pouches, followed by *on!* and *Rogue* nicotine pouch products. The slowest release rate was obtained for *Loop* nicotine pouches. In the profile region between 0 and 20 min, we observed a total nicotine percent release of 93% for *Zyn*, 87% for *on!*, 84% for *Rogue*, 71% for *Volt* and *Velo*, 66% for *Dryft*, and 48% for *Loop* nicotine pouch products. These observations indicate that despite the different manufacturers, nicotine content, and formulations; nicotine pouches can have similar nicotine release rate as shown in Fig. 1(b) from the overlapping release profiles for *Volt*, *Velo*, and *Dryft* pouches.

To further confirm these observations, the nicotine release profiles were analyzed by calculating the difference factor (f_1_) and similarity factor (f_2_) by adopting methodology referenced in the guidance for industry from FDA^14^. To achieve f_1_ and f_2_ calculations, *on!* nicotine pouches were used as the reference products and all other products were used as the test products (Table 3). For curves to be considered equivalent, f_1_ values should be close to zero and f_2_ values should be close to 100. Generally, f_1_ values up to 15 (0–15) and f_2_ values of 50 or greater (50–100) demonstrate equivalency of the two curves. Only *Zyn* and *Rogue* pouches were found to be equivalent to *on!*. All other pouches were found to be different than *on!* nicotine pouches.

**Table 3:** Calculated and estimated f_1_ and f_2_ values for six Nicotine pouch products compared to *on!* Nicotine pouches.

Products	Calculated from experimental data			Estimated data from First-Order Kinetics model		
	f_1_	f_2_	Equivalency	f_1_	f_2_	Equivalency
*on!* vs. *Zyn*	14.3	52.9	Yes	12.4	54.3	Yes
*on!* vs. *Velo*	14.5	47.3	No	16.5	45.5	No
*on!* vs. *Dryft*	22.6	38.2	No	25.0	35.9	No
*on!* vs. *Rogue*	2.9	81	Yes	3.2	80.4	Yes
*on!* vs. *Volt*	14.9	46.6	No	15.2	46.8	No
*on!* vs. *Loop*	34.4	27.8	No	47.2	21.6	No

**Fig. 2:**
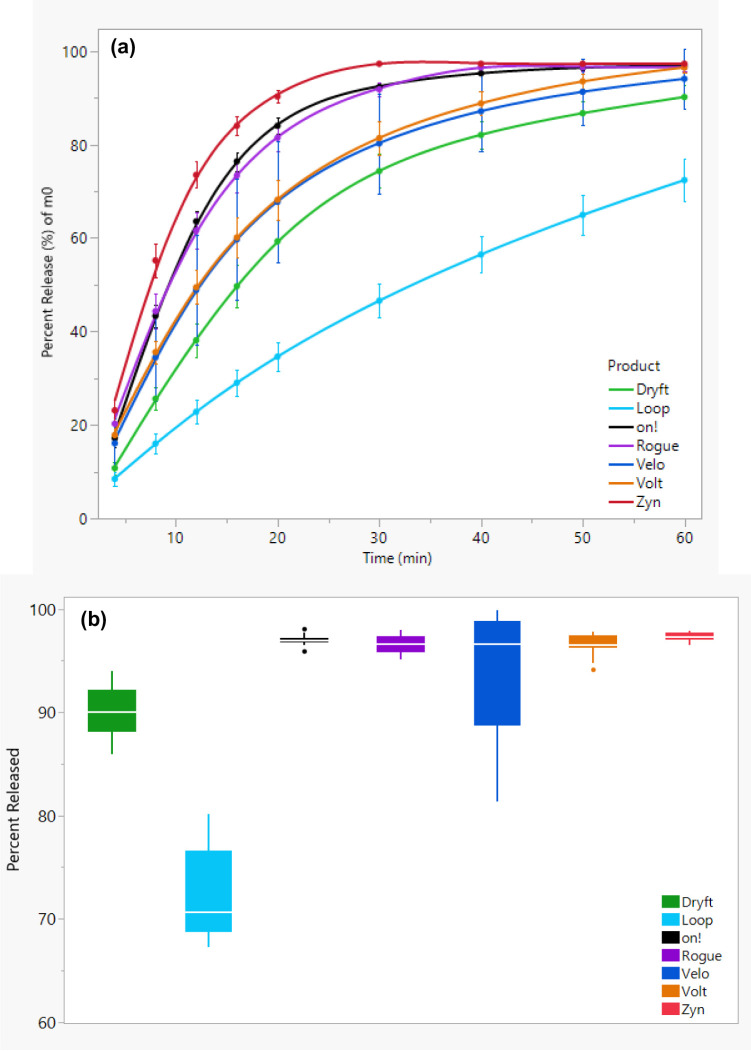
(**a**) Estimated percent of total nicotine dissolution release profiles obtained from the first-order kinetic model and (**b**) distribution of the estimated nicotine released at 60 min based on the nicotine level at *M*_*0*_.

To further validate the experimental dissolution data, we applied a first-order kinetics model to predict the nicotine content at *t*_∞_ and estimate the total amount of nicotine available for release within a 60-minute dissolution period. This modeling approach was previously described in the Materials and Methods section. The estimated nicotine content (mg/pouch) at *M*_*0*_ for each of the seven nicotine pouch products is summarized in Table 2: *on!* (8.380 mg/pouch), *Zyn* (5.350 mg/pouch), *Velo* (4.297 mg/pouch), *Dryft* (5.695 mg/pouch), *Rogue* (4.595 mg/pouch), *Volt* (8.225 mg/pouch), and *Loop* (4.740 mg/pouch). Furthermore, the percent of nicotine released for each individual pouch of the twelve replicates for each product from the total nicotine predicted at t_∞_ based on the cumulative nicotine release overtime is shown in Fig. 2(a). For this, the dissolution release profiles were normalized by calculating the percentage released at each time point. This percentage released was calculated by taking the amount of nicotine released at each time point for each fraction by the amount of nicotine at the beginning of the test, the *M*_*0*_ in the first-order kinetic model. The rate constant for each dissolution release profile was calculated using the average curve produced using Eq. 1. The rate constant was not calculated from each individual curve and therefore no average or standard deviation was calculated.

The estimation shows the fastest release rate for *Zyn* nicotine pouches, followed by *on!* and *Rogue* nicotine pouches. *Volt*, *Velo*, and *Dryft* exhibited comparable release rates to one another, but slower than *Zyn*, *on!*, and *Rogue*. The slowest release rate was obtained by *Loop* where the rate was slower than the other six nicotine pouch products. The distribution of the estimated nicotine released is shown graphically in Fig. 2(b). The estimated percent release was used to calculate the f_1_ and f_2_ factors in a similar fashion to the experimental data (shown in Table 3). The kinetic model results follow the same trend obtained from experimental data.

### Particle size and image analysis

**Fig. 3:**
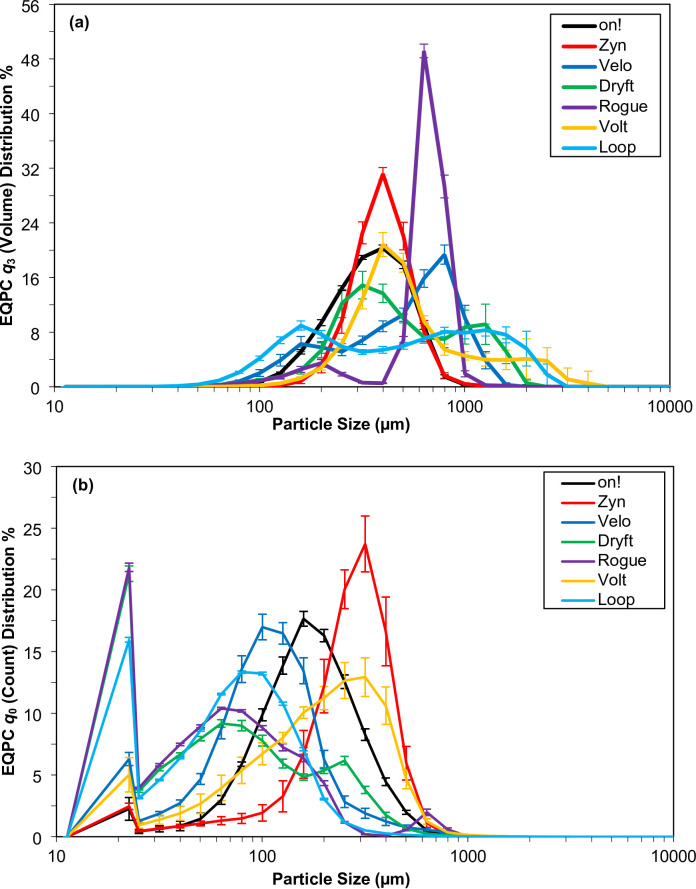
Dynamic image analysis particle size distribution (PSD) plots of the seven nicotine pouches. Distributions are calculated as the equal projection of a circle (EQPC) with both (**a**) volume-weighted (*q*_3_) and (**b**) count-weighted (*q*_0_) distributions (*n* = 12). Error bars represent one standard deviation (1 σ).

The particle size of the seven nicotine pouch products was determined by dynamic image analysis (DIA), optical microscopy, and scanning electron microscopy (SEM). The DIA results were calculated as the equal projection of a circle (EQPC) with results weighted by volume (*q*_3_) and particle count (*q*_0_). The particle size distributions (PSD) from dynamic image analysis are shown in Fig. 3 with the statistical results, the mean and intercepts at 10, 50, and 90% of the distributions (the *d*_10_, *d*_50_, and *d*_90_), summarized in Table 4. The intercepts of the distribution were used to calculate the span of each distribution curve using Eq. 2.

**Table 4:** Dynamic image analysis particle size distribution results of the seven nicotine pouches. The mean and intercepts at 10%, 50%, and 90% (*d*_10_, *d*_50_, and *d*_90_) are calculated as the equal projection of a circle (EQPC) with both volume-weighted (*q*_3_) and count-weighted (*q*_0_) results shown (*n* = 12). The error represents one standard deviation (1 σ).

Product	Particle size (µm)				Distribution span
	Mean	d_10_	d_50_	d_90_	
	Volume-Weighted (*q*_3_)				
*on!*	334.45 ± 6.30	167.20 ± 2.72	314.11 ± 4.66	515.85 ± 9.96	1.11 ± 0.02
*Zyn*	367.57 ± 13.40	233.18 ± 13.53	352.54 ± 11.72	511.95 ± 19.81	0.76 ± 0.02
*Velo*	503.85 ± 30.26	143.33 ± 12.01	494.91 ± 40.36	876.30 ± 47.10	1.49 ± 0.09
*Dryft*	537.65 ± 62.74	191.04 ± 14.22	399.79 ± 58.21	1121.36 ± 92.90	2.35 ± 0.18
*Rogue*	562.28 ± 12.34	248.44 ± 68.72	583.91 ± 5.64	753.03 ± 5.71	0.86 ± 0.12
*Volt*	658.83 ± 110.37	242.16 ± 9.87	430.56 ± 24.63	1543.75 ± 418.08	2.99 ± 0.85
*Loop*	626.74 ± 69.01	108.76 ± 2.81	444.42 ± 63.82	1468.33 ± 160.80	3.07 ± 0.16
	Count-Weighted (*q*_0_)				
*on!*	166.31 ± 4.84	66.70 ± 8.16	147.86 ± 3.70	292.63 ± 5.50	1.53 ± 0.07
*Zyn*	246.59 ± 14.53	99.75 ± 15.04	245.56 ± 16.90	383.52 ± 12.24	1.16 ± 0.09
*Velo*	110.16 ± 6.11	33.54 ± 2.66	92.94 ± 4.00	184.68 ± 14.58	1.62 ± 0.12
*Dryft*	91.32 ± 2.71	16.58 ± 0.16	57.22 ± 1.40	223.70 ± 7.28	3.62 ± 0.17
*Rogue*	80.83 ± 1.66	16.45 ± 0.08	52.69 ± 0.77	153.37 ± 1.68	2.60 ± 0.06
*Volt*	196.02 ± 16.86	44.85 ± 10.34	172.68 ± 21.72	370.39 ± 13.39	1.91 ± 0.24
*Loop*	73.81 ± 0.43	18.36 ± 0.09	63.01 ± 0.46	137.37 ± 0.83	1.89 ± 0.01

Based on the volume-weighted (*q*_3_) results (Fig. 3(a) and Table 4) two distribution types are clearly observed: *on!*, *Zyn*, and *Rogue* show mostly Gaussian-like distributions while *Velo*, *Dryft*, *Volt*, and *Loop* show mostly bimodal distributions. Both *on!* and *Zyn* are similar in particle size profiles with close mean values (334.45 μm for *on!* and 367.57 μm for *Zyn*) and similar distribution spans with *on!* being slightly larger at 1.11 than *Zyn* at 0.76. The mean value for *Rogue* was larger at 562.28 μm and a distribution span of 0.86. For all other products evaluated, the bimodal distributions are more variable (observed with percent relative standard deviation greater than 6%) with the mean particle size being biased to larger values of 503.85 μm for *Velo*, 537.65 μm for *Dryft*, 658.83 μm for *Volt*, and 626.74 μm for *Loop*. Larger differences in the distribution intercepts yielded larger distribution spans greater than 1.4 (1.49 for *Velo*, 2.35 for *Dryft*, 2.99 for *Volt*, and 3.07 for *Loop*). These results show the challenges in observing a direct relationship between the particle size distribution of the nicotine pouch products and the dissolution release rate due to other factors that could impact dissolution.

While volume-weighted particle size distributions can be skewed by a small number of larger particles, count-weighted distributions (Fig. 3(b) and Table 4) are able to detect smaller particles (fines) that are present in nicotine pouches. The lower limit of the lens used for all DIA measurements was 20 μm and all products had a large amount of fine in the first bin fraction (22.57 μm) ranging roughly from 2.5% for *on!* and *Zyn* to 22% for *Rogue* and *Dryft*. When further looking at the count-weighted results, there are two distinct groups with a *d*_50_ value above or below 100 μm. The above group includes *on!*, *Zyn*, and *Volt* at 147.86 μm, 245.56 μm, and 172.68 μm, respectively. The remaining products, *Velo*, *Dryft*, *Rogue*, and *Loop*, have *d*_50_ values at 92.94 μm, 57.22 μm, 52.69 μm, and 63.01 μm. The volume and count weighted data differences in nicotine pouch products’ filler indicate the presence of various particles and suggest the use of different granulation processes.

**Fig. 4:**
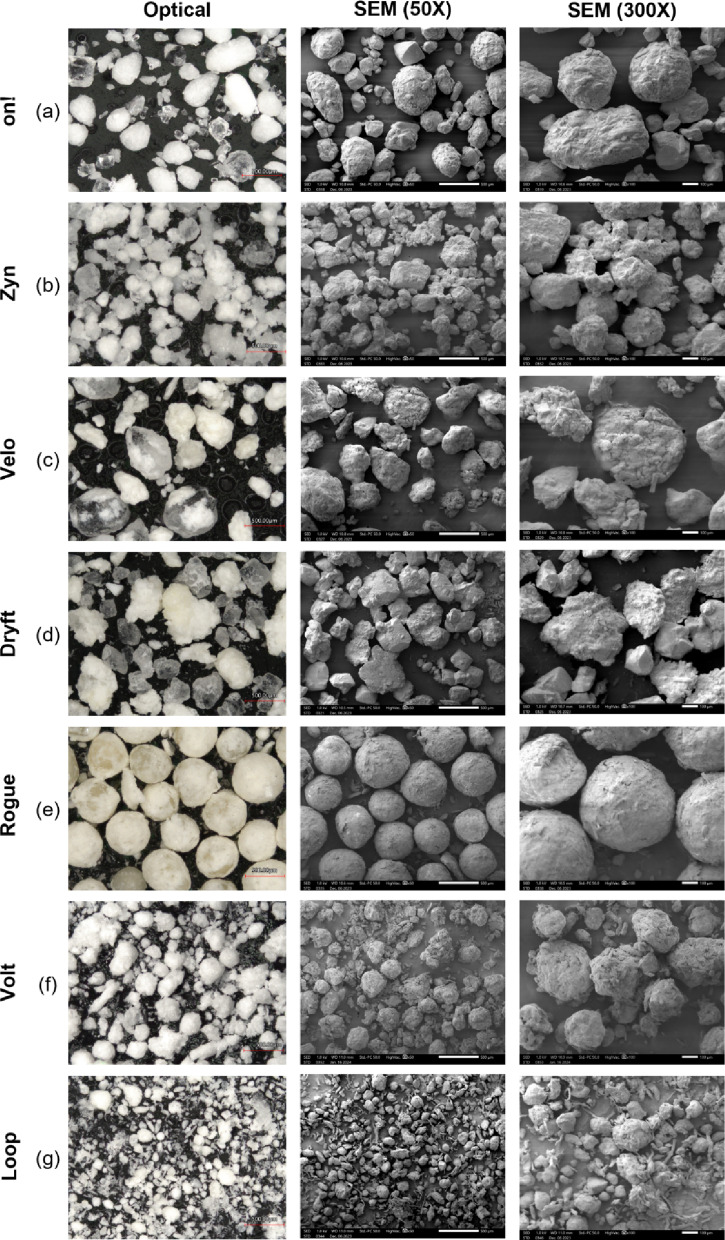
Optical and scanning electron microscopy (SEM) micrographs of the filler from the seven nicotine pouch products.

To gain additional information into the makeup of particles within the nicotine pouch fillers, optical microscopy and SEM were performed with the micrographs shown in Fig. 4. The average particle size obtained from the optical micrographs are summarized in Table 5. The differences from the DIA results can be accounted for by general differences in particle size techniques and calculations used to calculate mean size. Despite showing Gaussian-like distributions in the DIA measurements, the micrographs for *on!* and *Zyn* showed a variety of granulated particles that were both circular and elliptical in shape as well as crystalline material. The two classes of particles observed in the SEM micrographs for *Loop*, where the rough surface of the granulated particles is distinguished from the crystalline solid material. On the other hand, *Rogue* micrographs showed particles that were mostly granulated spheres of roughly the same size. Those particles that were not spheres were the debris from the granulated spheres.

**Table 5:** Filler particle size and pouch material pore size calculated using optical microscopy for the seven nicotine pouches. Error represent one standard deviation (1 σ).

Product	Optical microscopy particle size (µm)	Pouch material void pore size (µm)
*on!*	380.56 ± 73.52	49.219 ± 14.375
*Zyn*	343.42 ± 186.62	67.951 ± 20.323
*Velo*	329.56 ± 180.01	49.918 ± 13.890
*Dryft*	317.98 ± 81.89	52.730 ± 8.618
*Rogue*	595.47 ± 76.20	73.428 ± 17.330
*Volt*	296.88 ± 73.61	104.812 ± 34.138
*Loop*	138.27 ± 48.75	74.777 ± 21.905

The particle morphology observed using optical and SEM microscopy (Fig. 4) showed greater variation of particles for products with bimodal distributions in the DIA results. The optical microscopy images obtained for *Velo* indicated the presence of a combination of granulated and crystalline particles. However, the SEM micrographs show particles with rough and granulated surfaces. *Dryft* nicotine pouches show similar characteristics in addition to the presence of particles that are larger and irregular in shape. *Volt* particles were found to be a variety of granulated irregular spheres and larger agglomerates of multiple particles. *Loop* was dried prior to imaging (due to the high volatile content of the particles) leading to the largest variety of particles from all the studied nicotine pouch products with granulated and fibrous particles observed.

**Fig. 5:**
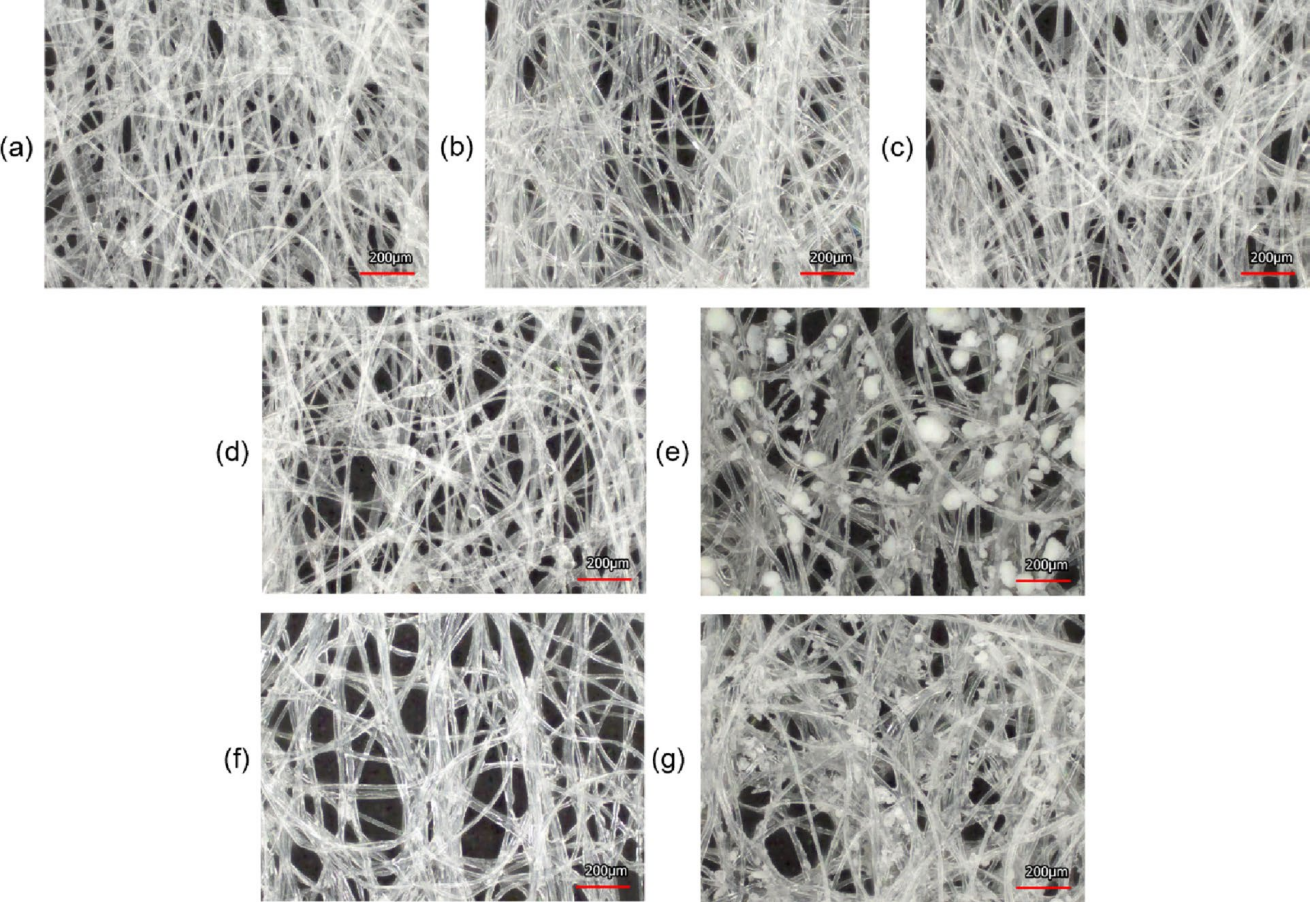
Optical imaging of outer pouch material of the seven nicotine pouch products for (**a**) *on!*, (**b**) *Zyn*, (**c**) *Velo*, (**d**) *Dryft*, (**e**) *Rogue*, (**f**) *Volt*, and (**g**) *Loop*.

The outer pouch material, the outer paper-like material that encases the filler, was also imaged by optical microscopy (Fig. 5) and the average pore size as measured by the void space of the image was calculated (Table 5). The pore size is related to the fiber density with *Volt* having the largest pore size of 104.812 μm. *Rogue* and *Loop* have similar pore size of 73.428 μm and 74.777 μm. *Zyn* and *Dryft* were next with 67.951 μm and 52.730 μm. The smallest pore size was observed for *on!* and *Velo* with 49.219 μm and 49.918 μm. These differences in pore size did not correlate with the dissolution rates where *Rogue* and *Loop* have similar pouch material pore size, but very different nicotine dissolution release profiles. In addition, *on!*, *Zyn*, and *Rogue* had different pore sizes yet equivalent nicotine dissolution release profiles.

### Density, flowability, and porosity measurements

**Fig. 6:**
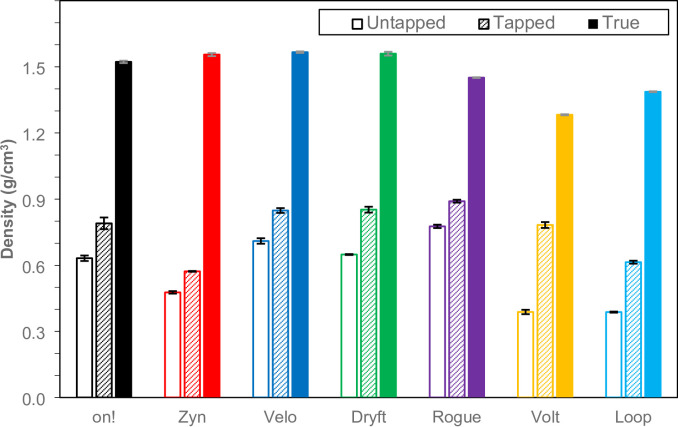
Density measurements of nicotine pouch filler in (g/cm^3^) where the hollow bars represent untapped bulk density, the dashed bars represent the tapped bulk density, and the solid bars are the true density as measured using a gas pycnometer. The results are the average of three replicates (*n* = 3) with the error bars representing one standard deviation (1 σ).

The density of the nicotine pouch products was measured using three different density methodologies: untapped bulk density (*ρ*_UB_), tapped bulk density (*ρ*_TB_), and true density (*ρ*_T_). Both the untapped and tapped bulk density measurements were performed following the United States Pharmacopeia (USP) method^23^. The true density was measured using gas pycnometry with helium gas^24–26^. The results for all measurements are displayed in Fig. 6 and summarized in Table 6. *Zyn*, *Loop*, and *Volt* have the lowest untapped bulk density at 0.4768 g/mL, 0.3882 g/mL, 0.3874 g/mL, respectively. The untapped bulk density for the other products were 0.6319 g/mL, 0.7110 g/mL, 0.6485 g/mL, and 0.7762 g/mL for *on!*, *Velo*, *Dryft*, and *Rogue*, respectively. The tapped bulk density values of *Zyn* and *Loop* at 0.572 g/mL and 0.614 g/mL, respectively, were lower compared to the other nicotine pouch products tested. In this case, the values for the tapped bulk density clustered together at 0.790 g/mL, 0.848 g/mL, 0.852 g/mL, 0.891 g/mL, 0.782 g/mL for *on!*, *Velo*, *Dryft*, *Rogue*, and *Volt*, in corresponding order. The true density values of *on!*, *Zyn*, *Velo*, and *Dryft* were similar at 1.523 g/mL, 1.555 g/mL, 1.566 g/mL, and 1.560 g/mL, respectively. The other true density values were 1.451 g/mL, 1.283 g/mL, and 1.387 g/mL for *Rogue*, *Volt*, and *Loop*, respectively.

**Table 6:** Experimental density results and calculated flowability and porosity values for nicotine pouch fillers. The untapped and tapped bulk density values were used to calculate the carr’s index and Hausner ratio flowability values. The tapped bulk density and true density were used to calculate the percent porosity. For all testing, an average of three replicates (*n* = 3) is shown with the error representing one standard deviation (1 σ).

Product	Density (g/mL)			Calculated values		
	Untapped (*ρ*_UB_)	Tapped (*ρ*_TB_)	True (*ρ*_T_)	Carr’s Index	Hausner Ratio	% Porosity
*on!*	0.6319 ± 0.0125	0.790 ± 0.026	1.523 ± 0.0053	20.02	1.250	48.109
*Zyn*	0.4768 ± 0.0057	0.572 ± 0.002	1.555 ± 0.0069	16.68	1.200	63.208
*Velo*	0.7100 ± 0.0124	0.848 ± 0.011	1.566 ± 0.0036	16.32	1.195	45.839
*Dryft*	0.6485 ± 0.0021	0.852 ± 0.013	1.560 ± 0.0088	23.91	1.314	45.359
*Rogue*	0.7762 ± 0.0077	0.891 ± 0.007	1.451 ± 0.0018	12.85	1.147	38.620
*Volt*	0.3882 ± 0.0100	0.782 ± 0.013	1.283 ± 0.0024	50.38	2.015	38.999
*Loop*	0.3874 ± 0.0025	0.614 ± 0.007	1.387 ± 0.0022	36.90	1.585	55.739

The Carr’s Index and Hausner Ratio both use the untapped and tapped bulk density values to give a measure of the flowability of the nicotine pouch filler using Eqs. 4 and 5. For the Carr’s Index, a value greater than twenty-five (25) indicates a poor flowing filler. Only *Volt* and *Loop* met this criterion with values of 50.38 and 36.90, accordingly. These findings align with the Hausner Ratio, which states that powders with a value greater than 1.25 are not free flowing; *Volt* had a ratio of 2.015 and *Loop* had a ratio of 1.585. There was a disagreement however with *Dryft* where the Carr’s Index was 23.91 and the Hausner Ratio was 1.314 which could be attributed to the filler surface properties.

The porosity of the nicotine pouch filler was calculated using the true density and tapped bulk density values using Eq. 6. The percent porosity is a measure of the total void space of open and closed pores in the nicotine pouch filler. The results varied from 38% to 63% showing differences in the structure and packing of the filler material. The product with the highest porosity was *Zyn* with a value of 63%; while *Rogue* had the lowest porosity with a value of 39%. This low value for *Rogue* would suggest denser and efficient packing of the nicotine pouch filler in addition to the larger particle size. These results are in agreement with the SEM images where the *Rogue* particles appeared as smooth spheres.

### Crystallinity by DSC

**Fig. 7:**
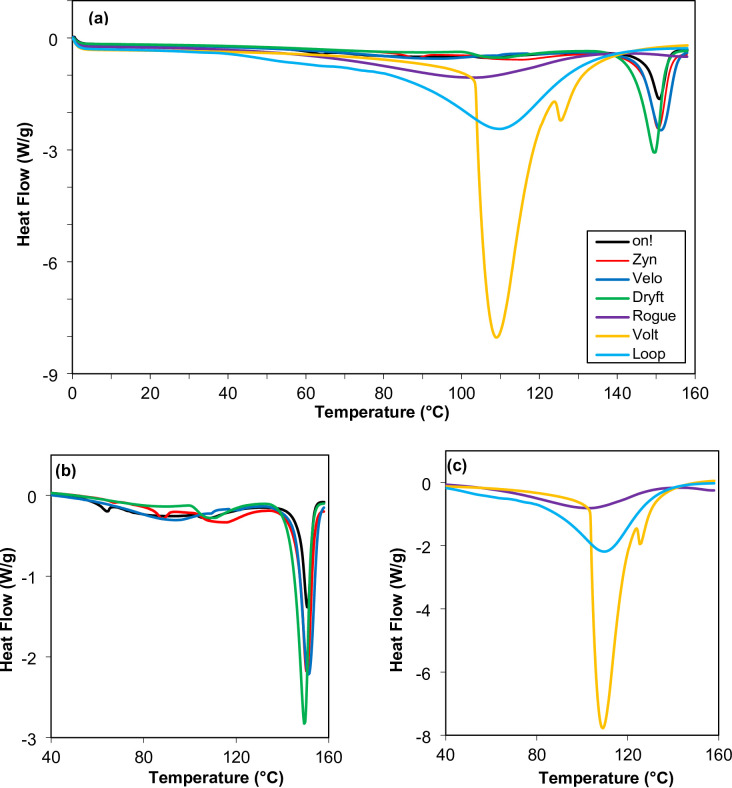
DSC thermograms of the seven nicotine pouch fillers tested where: (**a**) overlay for all products (**b**) thermograms of the high temperature melting point, and (**c**) thermograms of the low temperature melting point.

The differential scanning calorimetry (DSC) thermograms for the seven tested nicotine pouch products are summarized in Fig. 7. The higher the crystallinity, the higher the heat required to melt the material and therefore the higher the melting enthalpy^31^. Generally, the crystalline and ordered materials have a higher melting point than less crystalline material^32^. For the high crystalline material more energy is needed to vibrate the crystal lattice or polymer chain and break the ordered structure compared to materials with lower crystallinity. The size and shape of melting peaks along with the melting temperature in the DSC thermograms are useful to evaluate the crystallinity of a material. The thermograms were used to determine the melting enthalpy, onset temperature, and melting temperature due to major thermal transitions for all seven nicotine pouch fillers with the results summarized in Table 7. These results divide the products into two categories. The first category consists of crystalline fillers with high melting points (Fig. 7(b)), including *Zyn*, *on!*, *Dryft*, and *Velo*, which exhibit melting peaks around 150 °C. The second category consists of fillers with lower melting points and includes *Rogue*, *Volt*, and *Loop* (Fig. 7(c)). The crystallinity order based on enthalpy melting peaks for the first group is *Dryft* (99.85 J/g), *Velo* (84.89 J/g), *Zyn* (67.40 J/g), and *on!* (38.25 J/g). Among the second group, *Rogue* shows a weak, shallow melting peak at 102 °C, *Volt* exhibits a strong peak at 112 °C along with a smaller notch peak, and *Loop* displays a broad peak at 110 °C. The crystallinity order for this group, based on enthalpy melting peaks, is *Volt* (637 J/g), *Loop* (515 J/g), and *Rogue* (103 J/g). Among the seven tested nicotine pouch fillers, three, *on!*, *Zyn*, and *Velo*, possess a secondary melting peak in the 60–115 °C range. This secondary thermal transition might be due to the presence of water molecules and other additives used in the granulation or products formulation. Water and humectant-like additives might act as plasticizer and a glass transition temperature (*T*_g_) was observed at 104 °C, 105 °C, 83 °C, and 47 °C for *on!*, *Zyn*, *Velo*, and *Loop*, respectively. The *T*_g_ is the temperature at which an amorphous material changes from a hard or glassy state to a flexible state. This is not the melting point of a material^33^.

**Table 7:** DSC results of the seven nicotine pouch fillers with a temperature sweep of 0–160 °C at 10 °C/min with the major and secondary thermal transitions (where applicable).

Products	Major thermal transition			Secondary thermal transition			
	Onset Temp. (°C)	Enthalpy of Melting (Δ*H*, J/g)	Melting Temp. (°C)	Onset Temp. (°C)	Enthalpy of Melting (Δ*H*, J/g)	Melting Temp. (°C)	Glass Transition Temp. (°C)
*on!*	147.10	38.254	150.82	30.32	1.6572	63.93	104.19
*Zyn*	146.43	67.401	150.52	83.96	3.323	88.23	105.50
*Velo*	145.71	84.891	151.29	N/A	N/A	N/A	82.83
*Dryft*	144.10	99.852	149.58	101.03	13.205	110.39	104.39
*Rogue*	55.29	200.94	102.51	N/A	N/A	N/A	N/A
*Volt*	104.05	637.07	112.11	N/A	N/A	N/A	N/A
*Loop*	78.45	515.32	109.94	N/A	N/A	N/A	46.77

N/A – Not applicable.

There is a strong correlation between true density and crystallinity of the tested nicotine pouch fillers. Higher crystallinity typically leads to a higher true density due to the more ordered arrangement of particles. It does not necessarily lead to a higher tapped untapped bulk density, as the bulk density is also influenced by other factors such as particle size, shape, and packing efficiency. The first category of fillers, such as *Zyn*, *on!*, *Dryft*, and *Velo*, are crystalline fillers with high melting points and high true density. In contrast, the second category of fillers, including *Rogue*, *Volt*, and *Loop*, have low melting points and relatively low true density.

### Additional characterization analyses

To further characterize the nicotine pouch fillers, other chemical and physical characterizations were performed including solubility, elemental analysis, moisture content, pH of aqueous extracts, and viscosity of extracted nicotine pouch solutions. The nicotine pouch in artificial saliva percent solubles are summarized in Table 8. *Zyn* had the highest percentage soluble filler at 78.26% soluble while *Rogue* was the least soluble at 41.21%. The remaining products with *Dryft*, *on!*, *Velo*, and *Volt* ranked from most to least soluble at 68.03%, 66.35%, 64.41%, and 59.75%, respectively. *Loop* nicotine pouch filler could not be filtered and therefore no solubility results were obtained. Compared to the dissolution results, *Zyn* had both the fastest dissolution rate as well as the highest solubility. Although this relationship is present, it is not a direct correlation between solubility and dissolution. This is seen with *Dryft* which has the second highest solubility, but one of the slowest dissolution release rates. Another example would be *Rogue* which was equivalent to *on!* in dissolution rate but had the lowest solubility and roughly 25% more insoluble content compared to *on!* filler.

**Table 8:** Average percent soluble content, moisture content (as measured by halogen oven OV), pH, and water activity (a_W_) for the seven nicotine pouch fillers. Results are an average of three replicates (*n* = 3) with the error representing one standard deviation (1 σ).

Product	Percent soluble (%)	OV (% MC)	pH	Water activity (a_W_)
*on!*	66.35 ± 0.15	4.48 ± 0.20	8.62 ± 0.16	0.57 ± 0.004
*Zyn*	78.26 ± 0.77	3.52 ± 0.08	7.62 ± 0.01	0.56 ± 0.012
*Velo*	64.41 ± 2.67	6.62 ± 0.23	8.98 ± 0.05	0.56 ± 0.003
*Dryft*	68.03 ± 0.68	3.71 ± 0.15	8.31 ± 0.10	0.56 ± 0.001
*Rogue*	41.21 ± 0.35	14.70 ± 0.14	7.43 ± 0.17	0.65 ± 0.001
*Volt*	59.75 ± 2.54	37.18 ± 1.80	8.47 ± 0.02	0.92 ± 0.001
*Loop*	N/A	26.64 ± 0.84	8.62 ± 0.01	0.87 ± 0.001

N/A – Not applicable.

To better understand the solubility of these nicotine pouch products, elemental analysis was performed using SEM-EDS on the filler of these pouches with the results summarized in Table 9. For all nicotine pouch products, carbon and oxygen were the most abundant elements quantified. These results were expected since the filler of nicotine pouch products commonly uses microcrystalline cellulose (MCC) as a base. The variable amounts of sodium in nicotine pouch products may indicate differences in the use of pH adjusters and salts in the formulations. Other salts, including potassium and calcium, were found in *Zyn* and *Loop* (potassium) and in *Volt* (calcium). Silicon was only present in *Rogue* (4.59%) and *Loop* (2.01%) and could be due to the presence of an ingredient such as Talc.

**Table 9:** Elemental analysis of the seven nicotine pouch fillers of nicotine pouch products as measured by SEM-EDS spectra analysis.

Product	EDS spectra analysis								
	C	O	Na	Al	Si	Cl	K	Ca	Total
*on!*	56.85	41.85	1.30	ND	ND	ND	ND	ND	**100.00**
*Zyn*	49.60	49.46	0.83	ND	ND	ND	0.11	ND	**100.00**
*Velo*	52.06	44.89	3.05	ND	ND	ND	ND	ND	**100.00**
*Dryft*	47.00	50.16	2.84	ND	ND	ND	ND	ND	**100.00**
*Rogue*	42.44	49.05	2.03	ND	4.59	1.89	ND	ND	**100.00**
*Volt*	51.05	44.03	2.06	ND	ND	2.58	ND	0.28	**100.00**
*Loop*	48.99	47.70	0.98	BLOQ	2.01	BLOQ	0.12	BLOQ	**99.99**

ND – Not detected.

BLOQ – Below limit of quantitation.

Moisture content (% MC) was determined by measuring oven volatiles using a halogen oven at 80 °C for 10 min, with mass change recorded during the experiment (results shown in Table 8). *Zyn* (3.52% MC), *Dryft* (3.71% MC), *on!* (4.48% MC), and *Velo* (6.67% MC) had the lowest moisture levels. In contrast, *Rogue*, *Loop*, and *Volt* exhibited much higher moisture contents of 14.70% MC, 26.64% MC, and 37.18% MC, respectively. The moisture content and solubility are inversely proportional with the exceptions of *Volt* and *Loop* (where solubility could not be measured). The high moisture content of *Volt* could potentially explain the high enthalpy and low melting point observed in the crystallinity analysis.

The water activity (*a*_w_) of the seven nicotine pouch products showed results consistent with their moisture content, as summarized in Table 8. Two distinct groups were observed: *Zyn*, *Velo*, *Dryft*, and *on!* had water activity values below 0.6 (0.56 for *Zyn*, *Velo*, and *Dryft*; 0.57 for *on!*), while *Rogue* (0.65), *Loop* (0.87), and *Volt* (0.92) had values above 0.6.

 The pH of the aqueous extracts nicotine pouch fillers were measured using the CDC method for smokeless tobacco products with the results summarized in Table 8^28^. The pH of the tested products ranged from 7.43 for *Rogue* to 8.98 for *Velo*. No clear trend was observed between pH and nicotine content, solubility, or moisture content. This suggests that pH is primarily influenced by the overall formulation of the nicotine pouch products rather than these individual properties.

**Fig. 8:**
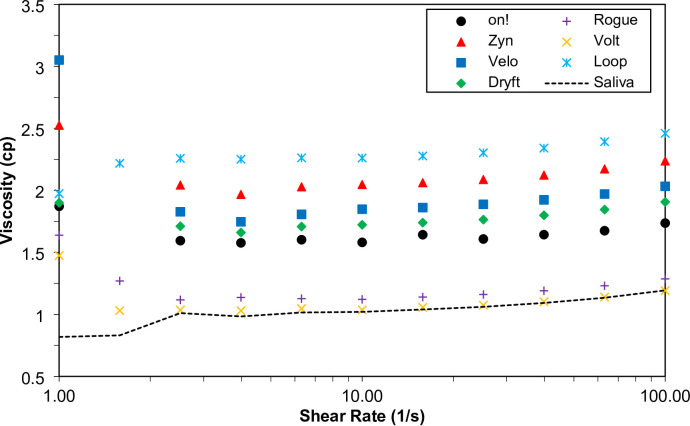
Viscosity measurements of the seven nicotine pouch extracted solutions measured using a concentric cylinder and temperature-controlled jacket at ambient temperature. Measurements were taken at intervals between 1–100 1/s shear rate and marked with points for each product. The dotted line is the viscosity of artificial saliva at the same shear rates for baseline.

The viscosity of the extracted solutions from nicotine pouch fillers in artificial saliva was measured at a sheer rate of 1–100 1/s (Fig. 8). These solutions contain the soluble portion of the nicotine pouch products. *Loop* exhibited the highest viscosity (2.2–2.3 cP). Because this solution could not be filtered during the solubility experiments, it is likely that insoluble components were present, forming a suspension^27^. The viscosity of the extracted solutions from the nicotine pouch fillers was found to be proportional to the solubility, with *Zyn* having a higher viscosity than the other products tested. *Rogue* and *Volt* were both the least soluble and least viscous (with the viscosity being slightly higher than the baseline of artificial saliva). The higher viscosity of *Loop* could be a contributing factor to the slow nicotine dissolution release rate.

## Discussion and conclusions

This study aimed to investigate the chemical and physical characterization of seven commercially available nicotine pouch products, a growing tobacco product category. The dissolution testing was carried out using a USP-4 flow-through cell apparatus, where dissolution profiles showed a faster release for *on!*, *Zyn*, and *Rogue* when compared to *Velo*, *Dryft*, *Volt*, and *Loop* nicotine pouches. When all nicotine pouches release profiles were compared to *on!*, only *Zyn* and *Rogue* were found equivalent based on f1 and f2 calculations. These results were confirmed with a first-order kinetic model.

The Gaussian-like particle size distributions for *on!*, *Rogue*, and *Zyn* nicotine pouches indicate more homogenous distribution of particles compared to the other products which displayed bimodal distributions. The morphology of filler particles and porosity of the outer pouch material were investigated by imaging using optical and scanning electron microscopy. While particle size does not appear to directly impact the dissolution rate, potential factors such as granulation processes and filler compositions could impact nicotine dissolution in different ways. The small and uniformly distributed particles observed in Gaussian-like distributions (associated with products having smaller span values) may reduce the rate of nicotine dissolution. The morphology and composition of the particles may also be a driver to the efficiency of nicotine dissolution within a nicotine pouch.

Using the bulk density results to estimate flowability, only *Volt* and *Loop* met the criteria to be considered poorly flowing filler. In an analogous manner, the porosity was estimated using the true density and tapped bulk density value with *Zyn* being the most porous filler (63%). This indicates that filler with high porosity can lead to consistent distribution within the pouch promoting faster nicotine dissolution.

Crystallinity was used to help understand the structural properties of the products with the products being grouped into high and low melting point groups (around 150 °C for high melting point and below 110 °C for low melting point). For the high melting point products, the crystallinity order (from highest to lowest) based on the enthalpy melting point was *Dryft*, *Velo*, *Zyn*, and *on!*. The crystallinity order for the low melting point products was *Volt*, *Loop*, and *Rogue*.

The crystallinity and melting point of nicotine pouch fillers appear to play a critical role in determining the dissolution and release rate of nicotine. In general, higher crystallinity is associated with slower nicotine release, whereas lower crystallinity facilitates faster release. This relationship is evident when comparing products across different melting point ranges. Products such as *Dryft*, *Velo*, *Zyn*, and *on!*, which exhibit higher melting points, demonstrate greater crystallinity. Within this group, *Dryft* and *Velo* show the highest crystallinity, followed by *Zyn* and *on!*. This trend aligns with dissolution observations, where *Dryft* and *Velo* exhibited slower release compared to *Zyn* and *on!*. Conversely, products like *Rogue*, *Loop*, and *Volt*, which melt at lower temperatures, generally indicate lower crystallinity and therefore faster dissolution. Among these, *Rogue* appears to be the least crystalline, supporting its rapid dissolution relative to *Loop* and *Volt*. Interestingly, despite their lower melting points, *Loop* and *Volt* display slower release than other pouches. This may be attributed to their classification as wet pouches, where moisture content and matrix structure could influence dissolution kinetics beyond crystallinity alone. These findings highlight the complexity of pouch design, where factors such as hydration state and filler composition interact with crystallinity to modulate nicotine release. From a formulation perspective, these observations underscore the potential for manufacturers to tailor nicotine delivery by adjusting crystallinity during processing.

Microcrystalline cellulose (MCC) is a major component of the crystalline structure of nicotine pouch fillers. Understanding and controlling the crystallinity of MCC is essential for optimizing nicotine release profiles. The amorphous regions of MCC are more hygroscopic, leading to higher moisture uptake. This moisture absorption can make the MCC softer and more flexible, facilitating the movement of nicotine molecules and potentially enhancing the release rate. By adjusting the MCC’s crystallinity, manufacturers can design products that deliver nicotine at desired rates, enhancing user satisfaction and product efficacy.

*Zyn* pouches contained the highest percent solubles in extracted saliva, while *Rogue* had the lowest. The higher soluble components can facilitate quicker nicotine release, as seen with *Zyn* pouches. Other factors aside from solubility, such as morphology and total active surface area (observed in this study as true density and porosity), contribute to the dissolution rate given the disparity between the solubility values of *Zyn* and *Rogue* despite similar dissolution profiles. *Volt* was found to have the highest percentage of moisture content and water activity while *Zyn* was the lowest for both. *Volt* and *Loop*, classified as wet pouch products, have higher moisture content and exhibited the slowest nicotine release profiles. This suggests that the presence of water and humectants in wet pouches may play a role in nicotine dissolution, a relationship worth further investigation.

The pH of aqueous extracts from the tested nicotine pouch products ranged from 7.6 to 9.0. While pH does not influence the release of nicotine from the pouch, it can affect absorption in the oral cavity. Higher pH levels increase the proportion of nicotine in its free (unprotonated) form, which is more readily absorbed through the oral mucosa, potentially leading to a faster onset of effects. This interaction also depends on the variable composition of saliva during use^29^. Importantly, these effects are separate from dissolution profiles, as *Zyn* and *Rogue* exhibited release rates comparable to *on!* and were among the fastest, despite having the lowest pH values. When testing the viscosity of the extracted solution, *Loop* was the most viscous, while *Volt* and *Rogue* had viscosities similar to artificial saliva. Higher viscosity can impede nicotine diffusion, leading to slower release rates, which explains why *Loop* never reached a plateau during dissolution testing. However, increased viscosity may alter the perception of mouthfeel or product experience, potentially influencing user satisfaction differently than the dissolution rate alone. This relationship between viscosity, nicotine release, and user perception represents an important area for future research.

The production of nicotine pouches involves various manufacturers utilizing different ingredients sourced from a variety of vendors. These ingredients can differ in their chemical composition and impurities. Additionally, manufacturers employ various granulation processes and use different materials for the pouches. As a result, a wide range of nicotine pouch products are created. Although all the tested products contain tobacco-derived nicotine, the combination of these factors lead to both similarities and differences in the physicochemical properties of the pouches. Consequently, multiple driving factors influence the nicotine release and overall performance of these pouches. By modifying the physical properties of the nicotine pouch products, manufacturers can design nicotine pouches with controlled nicotine release profiles. This study provides a comprehensive chemical and physical characterization of nicotine pouch products, offering valuable insights into their design and performance. Future studies should focus on controlled experiments using a single product formulation while systematically varying specific physical properties to understand their impact. While this work centers on product characterization, the methods and results presented here can inform development and manufacturing practices. It is important to note that changes occurring during actual use were beyond the scope of this study but represent an area for future investigation.

## Data Availability

Data sets generated during the study are available from the corresponding author upon request.
